# Orally Active Multi-Functional Antioxidants Delay Cataract Formation in Streptozotocin (Type 1) Diabetic and Gamma-Irradiated Rats

**DOI:** 10.1371/journal.pone.0018980

**Published:** 2011-04-26

**Authors:** James Randazzo, Peng Zhang, Jun Makita, Karen Blessing, Peter F. Kador

**Affiliations:** 1 Department of Pharmaceutical Sciences, University of Nebraska Medical Center, Omaha, Nebraska, United States of America; 2 Department of Ophthalmology, University of Nebraska Medical Center, Omaha, Nebraska, United States of America; Virginia Commonwealth University, United States of America

## Abstract

**Background:**

Age-related cataract is a worldwide health care problem whose progression has been linked to oxidative stress and the accumulation of redox-active metals. Since there is no specific animal model for human age-related cataract, multiple animal models must be used to evaluate potential therapies that may delay and/or prevent cataract formation.

**Methods/Principal Findings:**

Proof of concept studies were conducted to evaluate 4-(5-hydroxypyrimidin-2-yl)-N,N-dimethyl-3,5-dioxopiperazine-1-sulfonamide (compound **4**) and 4-(5-hydroxy-4,6-dimethoxypyrimidin-2-yl)-N,N-dimethyl-3,5-dioxopiperazine-1-sulfonamide (compound **8**), multi-functional antioxidants that can independently chelate redox metals and quench free radicals, on their ability to delay the progression of diabetic “sugar” cataracts and gamma radiation-induced cataracts. Prior to 15 Gy of whole head irradiation, select groups of Long Evans rats received either diet containing compound **4** or **8**, or a single i.p. injection of panthethine, a radioprotective agent. Compared to untreated, irradiated rats, treatment with pantethine, **4** and **8** delayed initial lens changes by 4, 47, and 38 days, respectively, and the average formation of posterior subcapsular opacities by 23, 53 and 58 days, respectively. In the second study, select groups of diabetic Sprague Dawley rats were administered chow containing compounds **4, 8** or the aldose reductase inhibitor AL1576. As anticipated, treatment with AL1576 prevented cataract by inhibiting sorbitol formation in the lens. However, compared to untreated rats, compounds **4** and **8** delayed vacuole formation by 20 days and 12 days, respectively, and cortical cataract formation by 8 and 3 days, respectively, without reducing lenticular sorbitol. Using *in vitro* lens culture in 30 mM xylose to model diabetic “sugar” cataract formation, western blots confirmed that multi-functional antioxidants reduced endoplasmic reticulum stress.

**Conclusions/Significance:**

Multi-functional antioxidants delayed cataract formation in two diverse rat models. These studies provide a proof of concept that a general cataract treatment focused on reducing oxidative stress instead of a specific mechanism of cataractogenesis can be developed.

## Introduction

Cataract is a major worldwide health care problem that especially affects the elderly. It is the leading cause of preventable blindness in the world, especially in developing countries in Africa and Asia [1], [2]. Currently, the only treatment for cataracts is the surgical removal of the opaque lens. With the worldwide aging population expected to increase four-fold [3], age-related cataracts are expected to become a major public health problem in terms of both increasing the number of required surgeries and the financial burden associated with these surgeries. Therefore, the development of a nonsurgical treatment for cataracts would have a profound beneficial impact not only on human health but also healthcare costs [1], [4], [5]. In fact, it has been estimated that a 10 year delay in cataract formation could reduced the need for cataract surgery by 50% [6]. Therefore the development of an efficacious anti-cataract drug would have a beneficial impact on both human health and healthcare costs.

Cataracts is a multi-factorial disease with important risk and/or modifying factors such as race, heredity, smoking, UV exposure, nutritional inadequacies, diabetes, and aging all contributing to cataract formation [5], [7], [8]. A common factor in most cataracts is oxidative stress [7], [8]. As within the lens, there is an age-dependent decrease in antioxidant defenses and an increase in the accumulation of redox-active metals such as iron and copper [9]. This results in hydrogen peroxide being able to interact with free iron to form cytotoxic hydroxyl radicals via the Fenton reaction.

A major impediment in the development of anti-cataract drugs is the lack of a specific animal model that mirrors the biochemical, physiological, and oxidative mechanisms observed in man. Therefore, the development of potential anti-cataract drugs requires their evaluation in multiple diverse animal models. For example, radiation of various intensities can induce cataracts in rats as a result of lens crystallin oxidation by free radicals/reactive oxygen species (ROS) [10]. Exposure to at least 2 Gy of gamma radiation has been documented to result in cataract formation after a dose-dependent latency period [11], [12], [13]. The deleterious effect of gamma rays on the lens is mediated by the radiolysis of water to hydrogen peroxide (H_2_O_2_) and hydroxyl radicals (OH^•^) in an aqueous environment and the formation of superoxide (O_2_^−•^) in an aerobic environment [14]. Of the ROS generated, hydroxyl radicals are the most reactive and they are considered to be the primary initiators of oxidative damage. Superoxide and hydrogen peroxide typically lack sufficient reactivity to oxidize critical biological macromolecules [15]. However, in the presence of redox reactive metals such as iron hydrogen peroxide and superoxide may undergo the Fenton and Haber-Weiss reactions that produce the stronger hydroxyl radicals [16]. Gamma radiation-generated superoxide has also been shown to cause the release of iron from ferritin, which can enhance the production of hydroxyl radicals that lead to DNA, protein, and lipid damage [14].

Another commonly used cataract model is the diabetic rat which has been established to develop sugar cataracts due to the aldose reductase (AR) catalyzed reduction of glucose to sorbitol [17], [18]. The intracellular accumulation of sorbitol results in hyperosmotic effects that can induce cellular swelling that initiates a cascade of biochemical steps that results in lens opacification. Among these steps is the osmotic induction of endoplasmic reticular (ER) stress [19], [20] which then can initiate an unfolded protein response (UPR) that generates ROS and apoptotic signaling. This generation of ROS has been reported by a number of investigators [21], [22], [23], [24].

**Figure 1:**
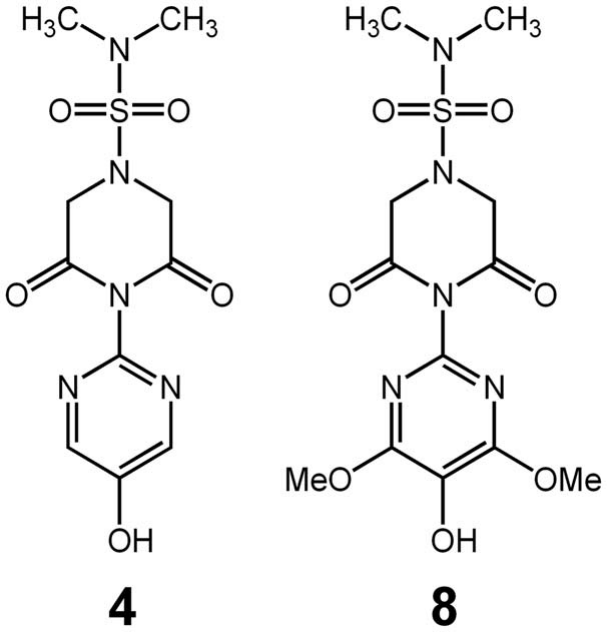
Structure of multi-functional compounds 4 (left) and 8 (right).

Recently, we have synthesized a novel series of compounds that include 4-(5-hydroxypyrimidin-2-yl)-N,N-dimethyl-3,5-dioxopiperazine-1-sulfonamide and 4-(5-hydroxy-4,6-dimethoxypyrimidin-2-yl)-N,N-dimethyl-3,5-dioxopiperazine-1-sulfonamide (Figure 1), that contain both a unique 2-amino-5-hydroxy-1,3-pyrimidine radical scavenging system and a 2,6-dioxipiperazine moiety that is able to chelate redox reactive metal ions such as iron and copper [25]. These compounds, referred to as compounds **4** and **8**, respectively, in the previous publication, are called multi-functional antioxidants because their radical scavenging and chelation ability are independent of each other. In cultured human lens epithelial cells (hLECs), these compounds reduced ROS generated both by either hydrogen peroxide or the Fenton reaction (iron and hydrogen peroxide) [25]. Here we demonstrate that oral administration of these compounds reduces the ROS-initiated progression of cataracts associated with gamma irradiation or osmotic stress in rats.

## Materials and Methods

### Materials

All reagents used were of reagent grade. Multi-functional antioxidants 4-(5-hydroxypyrimidin-2-yl)-N,N-dimethyl-3,5-dioxopiperazine-1-sulfonamide (**4**) and 4-(5-hydroxy-4,6-dimethoxypyrimidin-2-yl)-N,N-dimethyl-3,5-dioxopiperazine-1-sulfonamide (**8**) (99+% pure by HPLC) were synthesized as previously described [25]. The aldose reductase inhibitor, spiro-(2,7-difluoro-7H-fluorene-9,4′-imidazolidine)-2′-5′-dione (AL1576), was obtained from Alcon Laboratories (Fort Worth, TX). Anti-glucose-regulated protein 78/binding immunoglobulin protein (GRP78/Bip) was obtained from Abcam, Inc. (Cambridge, MA) and used at a dilution of 1∶2000. Chemiluminescent reagents and pre-stained and biotinylated protein ladders were obtained from Cell Signaling Technology (Danvers, MA). 4–15% Tris-HCl Ready Gel Precast gels and Trans-Blot® nitrocellulose membrane were obtained from Bio-Rad Laboratories (Hercules, CA). Nova Max Blood Glucose Monitoring system was obtained from Nova Biomedical (Waltham, MA). Bayer A1cNow test kits for glycosylated hemoglobin (HbA1c) were purchased from Bayer Health Care LLC. (Tarrytown, NY).

### Animal Care

All procedures were performed in strict accordance with the recommendations in the Guide for the Care and Use of Laboratory Animals of the National Institutes of Health and approved by the Institutional Animal Care and Use Committee of UNMC (Permit Number: 05-102-01).

### *In vivo* Irradiated Studies in Rats

Whole head gamma irradiation was conducted on 24 male pigmented Long Evans (150–200 g) rats. Prior to irradiation, these rats were randomly divided into 4 equal groups with Group 1 receiving standard rodent diet (untreated), Group 2 receiving standard rodent diet and the radioprotective agent pantethine, Group 3 receiving standard rodent diet supplemented with 0.025 wt% of compound **4**, and Group 4 receiving standard rodent chow supplemented with 0.025 wt% of compound **8**. Chow based treatments were initiated 14 days prior to whole head gamma irradiation and were continued until the study was terminated. Pantethine (1 g/kg i.p.) was administered 45 minutes prior to irradiation as described by Clark [26]. Each rat was placed in a standard plastic rat restraint and received 15 Gy of whole head gamma radiation (6000 Curie Cobalt-60 Source, UNMC Experimental Radiation Facility) at a rate of 0.341 Gy/min while unanesthetized. Following irradiation, each group of rats were housed three to a cage and provided water ad libitum. Because radiation reduces salivary gland function and causes dental problems, standard rodent chow was replaced with Nutra-Gel Diet™ supplemented with/without compounds **4** or **8** (Bio-Serv, Frenchtown, NJ) for the first 45 days post-irradiation. The groups were then returned to standard rodent diet with/without supplementation with compounds **4** or **8**. Body weight and food intake was monitored at 3–4 day intervals. Lens changes, monitored weekly by portable slit lamp following dilation with 1% Tropicamide, were evaluated as described by Livesy et al as follows –0: Clear; 1: Posterior subcapsular (PSC) haze; 2: Spokes, suture enhancement and powdery PSC deposits; 3: Prominent PSC punctate opacities with spokes and suture enhancement; 4: PSC opacity blocks appearance of retinal vessels; 5: Start of cortical involvement; and 6:Total lens opacity, visible with naked eye [27]. At the end of the study, all rats were euthanized by carbon dioxide asphyxiation, and their eyes were enucleated. The lenses were then carefully removed from the enucleated eyes by a posterior approach and frozen on dry ice for subsequent analysis.

### *In vivo* Diabetic Studies in Rats

Diabetes was induced in young (100 g) Sprague Dawley rats by tail vein injection of streptozotocin (75 mg/kg i.v.) as previously described [28]. Rats with blood glucose levels greater than 300 mg/dL were considered diabetic and divided equally into four groups of eight each. The first diabetic group received standard rat diet (untreated), the second and third diabetic group received standard rodent chow supplemented with either 0.05 wt% of compound **4** or **8**, and the fourth diabetic group received standard rodent chow containing 0.0125 wt% of the aldose reductase inhibitor (ARI) AL1576. Age-matched, non-diabetic rats receiving standard rodent chow were used as controls. Diets supplemented with compounds **4**, **8** or AL1576 were initiated five days following the initial streptozotocin injection and were continued until the end of the study. Rats were housed two to a cage and water was provided ad libitum. Body weight and food intake were monitored every 2–4 days. Lens changes in the rats were monitored by portable handheld slit lamp (Kowa Co Ltd., Tokyo, Japan) following mydriasis with 1% tropicamide at 3–4 days intervals. Lens opacification was evaluated on a scale of 0 to 3 [29]. Cataract progression of was scored as follows: 0, clear; 1.0, appearance of some equatorial vacuoles; 1.2, peripheral equatorial vacuoles; 1.4, obvious vacuole formation in <50% of lens; 1.6, obvious vacuole formation in >50% of lens; 1.8, total lens covered with vacuoles; 2.0, start of cortical opacity formation; 2.2, posterior capsule still visible by slit lamp; 2.4, easily detectable cortical opacity; 2.6, cortical opacity expanded to entire lens; 2.8, lens appears totally white; and 3, mature. Blood glucose levels at the start and end of the study were evaluated with a Nova Max blood glucose meter (Nova Biomedical, Waltham, MA). At the end of the study, rats were euthanized by carbon dioxide asphyxiation, blood was collected via cardiac puncture, and the eyes were enucleated. The lenses were carefully removed by a posterior approach from the enucleated eyes and frozen on dry ice for subsequent analysis.

### Lenticular Levels of Compounds 4 and 8

Lenses from diabetic and irradiated rats were homogenized in ground glass homogenizers with 2 mL of PBS containing 0.4 mM N,N-Dimethyl-4-(pyrimidin-2-yl)piperazine-1-sulfonamide as the internal standard. The homogenates were centrifuged for 5 min at 15,000 *g* at 4°C. Protein concentration in an aliquot of each supernatant was determined as previously described [30]. The remaining supernatant was then deproteinized with equal volumes of 0.3 N zinc sulfate and 0.3 N barium hydroxide [31]. Following an additional centrifugation at 15,000 *g* for 30 minutes at 4°C, the supernatant was transferred to a clean test tube and dried *in vacuo*. The remaining solid residue was suspended in HPLC grade chloroform and filtered through a 0.45 micron filter to remove particulates. The filtrate was again dried *in vacuo* and the remaining organic soluble residue containing both the internal standard and either extracted compound **4** or **8** was dissolved in HPLC grade acetonitrile. Samples (20 µL) were analyzed by an automated Hewlet Packard 1100 HPLC (Agilent Technologies, Santa Clara, CA) equipped with a Luna 5 µm C18, 250×4 column (Phenomenex Inc., Torrance, CA) at 25°C by elution with 50% aqueous methanol. The eluted compounds were monitored spectrophotometrically at 247 nm with a diode array detector and by ESI-MS (Thermo Finnigan LCQ; Thermo Fisher Scientific, Waltham, MA). Samples were quantified against constructed UV and ESI-MS standard curves of compounds **4** and **8**. All analyses were conducted in triplicate.

### Determination Lenticular Sorbitol Levels

Individual lenses from each of the *in vivo* groups were homogenized in a ground glass homogenizer containing 2 mL of PBS and three micromoles of the internal standard, xylitol. An aliquot of each lens homogenate was removed for protein quantification [30]. The remaining homogenate was then deproteinized by overnight centrifugation in YM-10 Microcon centrifugal filters (Millipore, Billerica, MA) at 8°C. Each filtrate was then dried *in vacuo*, dissolved in pyridine, and derivatized with phenyl isocyanate at 55°C for 60 min. After cooling, the reaction was halted by the addition of ice cold methanol. The derivatized samples (5 µL) were analyzed by reverse-phase HPLC on an automated Hewlet Packard 1100 (Agilent Technologies, Santa Clara, CA) equipped with a 150×4.6 mm TSK-GEL ODS-80Tm column (Tosoh Bioscience LLC, King of Prussia, PA) at 35°C. Samples were isocratically eluted with 20 mM potassium phosphate/acetonitrile (35∶65 v%) buffer, pH 7.0, at a flow rate of 1.0 ml/min. Samples were detected at 235 nm and quantified against standard curve of sorbitol (0.008–6.0 µmol).

### *In Vitro* ‘Diabetic’ Lenses Culture Studies

Young (100 g) Sprague Dawley rats were euthanized by carbon dioxide asphyxiation and their eyes were enucleated. Each lens was carefully removed by a posterior approach and placed in 2 mL of sterile TC-199 - bicarbonate media containing 30 mM fructose and 2% penicillin-streptomycin (culture media). To ensure that the lens was not damaged during dissection, each lens was first incubated for 4 hrs in a humidified incubator under 95% air and 5% CO_2_ at 37°C and checked for clarity [32]. All clear lenses were then equally divided into five groups and incubated for an additional 24 hrs as follows: Group 1: Culture media containing 30 mM fructose; Group 2: Culture media containing 30 mM xylose; Group 3: Culture media containing 30 mM xylose +0.1 mM AL1576; Group 4: Culture media containing 30 mM xylose +0.1 mM compound **4**; and Group 5: Culture media containing 30 mM xylose +0.1 mM compound **8**. After 24 hours, each lens was examined for morphological changes and the lens capsule containing the epithelial cells and superficial cortical fibers was removed and immediately frozen for subsequent analysis.

### Endoplasmic Reticulum Stress Determination

The thawed capsules from the cultured lenses were homogenized in ground glass homogenizers containing a mixture of RIPA buffer (Cell Signaling Technology, Danvers, MA) and Halt Protease and Phosphatase Inhibitor Cocktails (VWR International, West Chester, PA). Total protein concentrations were determined according to Bradford [30]. Equal protein samples were prepared in SDS-PAGE sample buffer and loaded onto precast polyacrylamide gels. Following electrophoresis, the separated proteins were transferred onto nitrocellulose membranes, blocked with 5% nonfat dry milk in TBS containing 0.1% Tween-20 (TBST), and incubated overnight at 4°C with primary antibodies for ER Stress (GRP78/Bip) and internal loading control (GAPDH). The membranes were washed three times with TBST and incubated with a secondary antibody conjugated with horseradish peroxidase (HRP) for 1 hour. The specific protein bands were visualized by incubating the membrane with luminol reagent and exposing the membrane to X-ray film. After digitizing the x-ray film image, the protein levels were quantified using the NIH ImageJ program (http://rsbweb.nih.gov/ij/). Comparative expression of GRP78/Bip was assessed by normalizing the total GRP78/Bip pixel volume (pixel density X area) to that of the respective GAPDH band.

### Statistical Analyses

Calculations were conducted using Microsoft Excel 2007 (Microsoft, Redmond, WA). Statistical analyses (ANOVA) were conducted using OriginPro® software version 8.1 (OriginLab Corp., Northampton, MA). Significant differences were defined as having a p<0.05.

## Results

### Efficacy of Compounds 4 and 8 on Cataract Formation in Gamma-irradiated Pigmented Rats

To determine if the multi-functional antioxidants can protect against radiation-induced cataracts, pigmented Long Evans rats received 15 Gy of gamma radiation (Cobalt 60 source) to the whole head, an amount established to result in 100% incidence of cataract development in albino animals [27], [33]. Twenty four 150–200 g Long Evans rats were split into four equal groups and all rats in each group received whole head radiation. Group 1 received standard rodent diet (untreated control). Group 2 also received standard rodent diet, but received pantethine, a known radioprotectant, by intraperitoneal injection 45 minutes prior to irradiation (1 g/kg) as previously described [26]. Groups 3 and 4 were administered standard rodent diet supplemented with either 0.025 wt% of compound **4** or **8** for two weeks prior to whole head irradiation and continued for the duration of the study.

**Figure 2:**
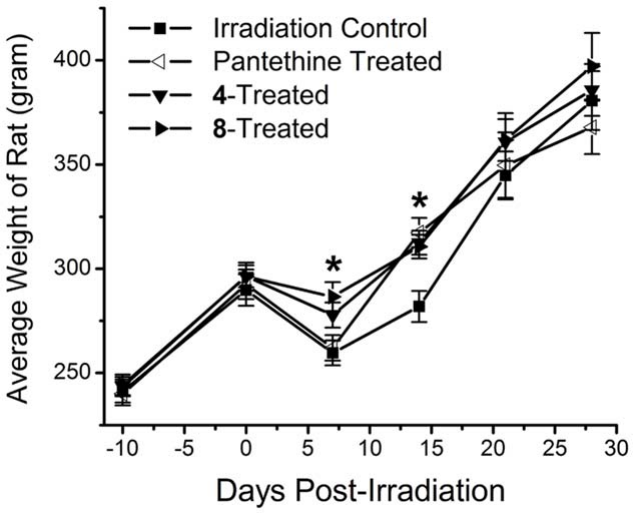
Comparison of weights of untreated, 4-treated, 8-treated, and pantethine-treated Long Evans rats post-irradiation. Treated rats weighed significantly more than untreated, irradiated rats. Mean ± S.D.; n = 6 rats. An asterisk (*) denotes a significant difference (p<0.05) when compared to untreated, irradiated rats.

It is established that whole head radiation results in a temporary loss of salivary gland function and dental problems. Therefore, all rats immediately received Nutra-Gel Diet™ supplemented with/without compounds **4** or **8** (Bio-Serv, Frenchtown, NJ) after 15 Gy of whole head gamma radiation (UNMC Experimental Radiation Facility). This diet is a nutrient and mineral equivalent “soft” diet designed for these types of experiments. Body weights were monitored to determine the effect of radiation on the rat's ability to feed. One week following radiation, untreated and panthethine-treated rats lost 10% of their body weights while similar rats treated with either compound **4** or **8** only lost 4%, suggesting that there was an improvement in the overall health in the multi-functional treated rats (Figure 2). After 14 days the body weights in the pantethine-treated group increased to the levels of the **4**- and **8**-treated irradiated rats had similar, enhanced body weights compared to untreated, irradiated control and by 21 days, all groups displayed similar weights. No difference in body weights was observed after 21 days post-irradiation (Figure 2). After salivary gland function returned and incisors regrew, all groups were returned to the solid standard rodent diet supplemented with/without compounds **4** or **8**. Feed records indicated that rats in groups 3 and 4 ingested an average dosage ± S.D. (mg/kg/day) of 25.0±1.5 for compound **4** and 26.6±1.6 for compound **8** prior to irradiation. Post irradiation, rats ingested an average of 17.5±5.5 mg/kg/day (mean ± S.D.) of compound **4** and 18.2±5.8 mg/kg/day (mean ± S.D.) of compound **8** as the weight percent supplementation remained constant.

**Figure 3:**
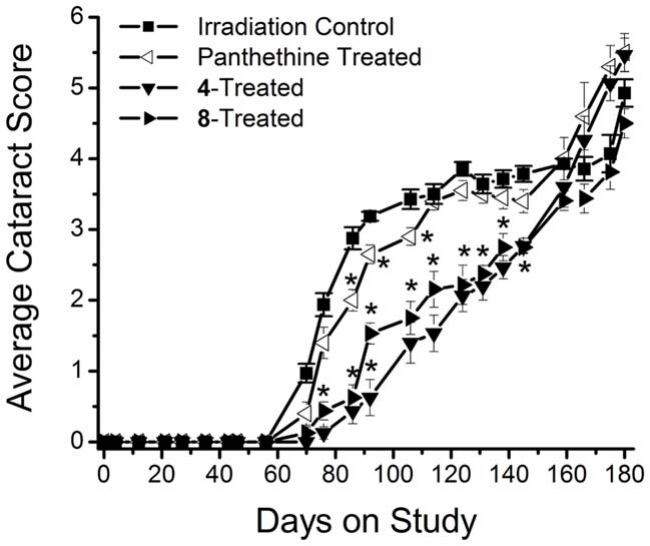
Progression of radiation-induced cataract formation. Figure illustrates a comparison of cataract score observed in whole head, gamma-irradiated rats treated with/without the radioprotectant, pantethine, or multi-functional antioxidants **4** and **8**. Values represent mean ± SEM; n = 6. An asterisk (*) denotes a significant difference (p<0.05) when compared to untreated, irradiated rats.

**Figure 4:**
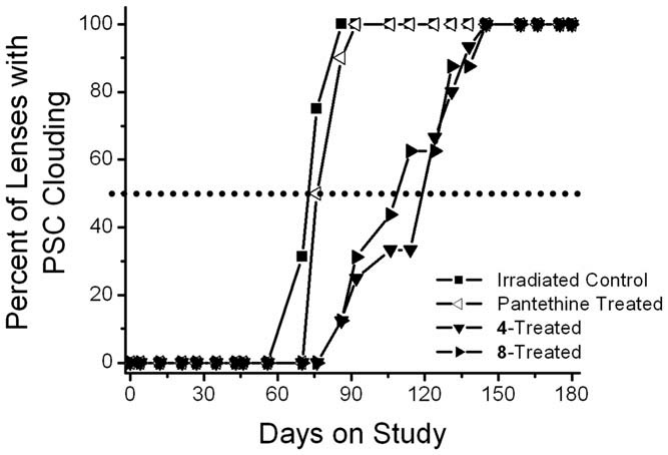
Percentage of animals progressing to PSC clouding (cataract score of 1.5) in gamma-irradiated rats treated with/without radioprotectant, pantethine, or multi-functional antioxidants 4 and 8. Values represent percentage of the 6 animals in each group. Delay was calculated based on the number of days required for 50% of the population to achieve this level of lens opacity (dotted line).

**Figure 5:**
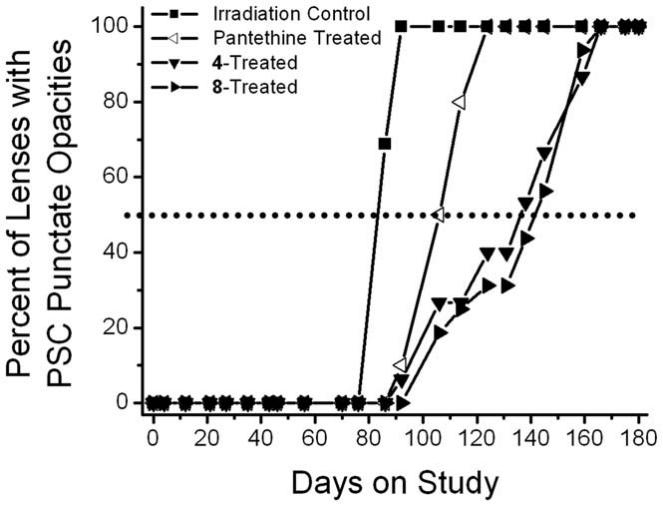
Percentage of animals progressing to prominent PSC punctate opacities (cataract score 3) in gamma-irradiated rats treated with/without radioprotectant, pantethine, and multi-functional antioxidants 4 and 8. Values represent percentage of the 6 animals in each group. Delay was calculated based on the number of days required for 50% of the population to achieve this level of lens opacity (dotted line).

The progression of radiation-induced cataracts has been described and subjectively monitored using a scale of 0 and 6 [27]. In the present studies, lens changes were moitored weekly by slit lamp following tropicamide mydriasis. As illustrated in Figure 3, initial lens changes appeared *ca.* 65 days after irradiation as a diffuse posterior subcapsular (PSC) clouding (haze). This was followed by the appearance of spokes, suture accentuation, and a progressive increase in PSC opacification (*ca*. 75 days) that resulted in the appearance of punctate opacities (*ca*. 90 days) that gradually increased in density so that visualization of the retina became difficult (*ca*. 150 days). Eventually by 180 days the entire lens became opaque, but mature cataracts were not found. Treatment with pantethine and multi-functional antioxidants **4** and **8** delayed the appearance of these lens opacities; however, the effect of pantethine was minimal. Cataract delay was assessed by calculating the time required for 50% of each group to develop PSC clouding or prominent PSC punctate opacities (Figures 4, 5). Compared to untreated irradiated rats, treatment with pantethine, **4** and **8** delayed the initial formation of PSC haze by 4, 47 and 38 days, respectively, and delayed the average formation of prominent PSC punctate opacities by 23, 53 and 58 days, respectively.

**Figure 6:**
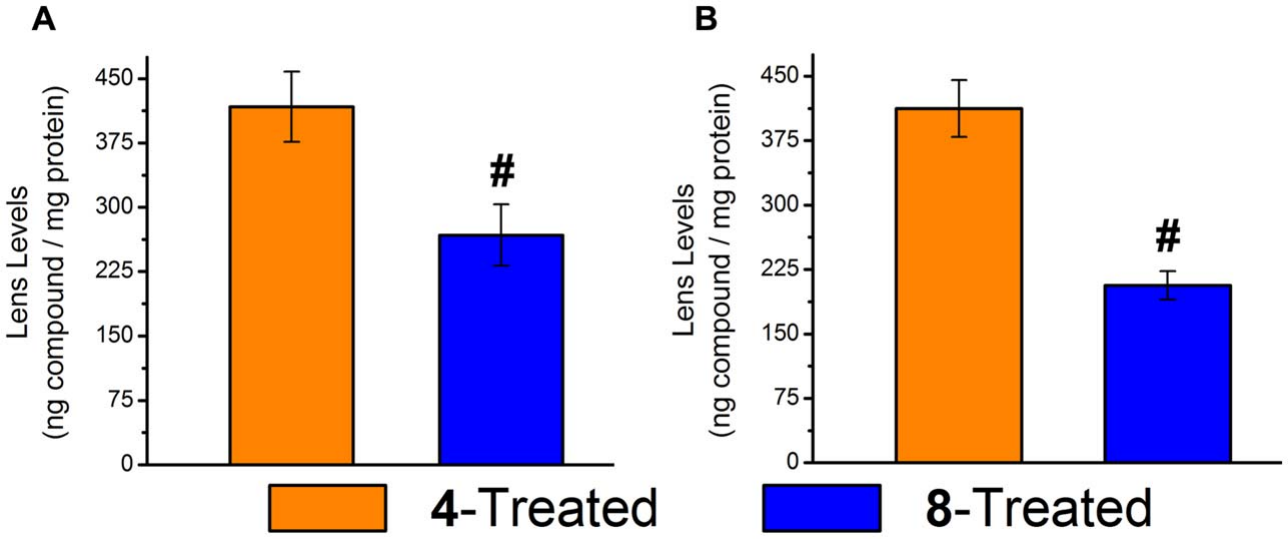
Lenticular levels of investigational compounds 4 and 8. **A** Accumulation in the irradiated lens after 28 weeks of feeding. **B** Similar accumulation of compounds **4** and **8** in the diabetic lens following feeding of diet supplemented with 0.05% compounds **4** and **8** for 7 weeks. Mean ± S.E.M.; n = 6. A pound sign (#) denotes a significant difference (p<0.05) when compared to the **4**-treated group.

At the end of the study, the lenses were analyzed for the amounts of compounds **4** and **8** present in the avascular lens. Analysis by HPLC-MS indicated that 417±41 ng/mg protein of compound **4** and 267±36 ng/mg protein of compound **8** were present in the irradiated lenses (Figure 6A).

### Efficacy of Compounds 4 and 8 on Delaying Diabetic ‘Sugar’ Cataracts

**Figure 7:**
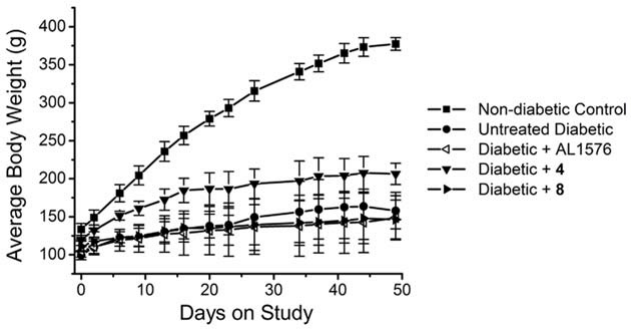
Average weights of non-diabetic control and untreated, 4-treated, 8-treated, and AL1576-treated diabetic Sprague Dawley rats. Mean ± S.D.; n = 8 rats.

While it is established that diabetic cataracts are initiated by the aldose reductase (AR) catalyzed accumulation of sorbitol, antioxidants have been reported to delay diabetic cataract formation. Therefore, multi-functional compounds **4** and **8** were evaluated for their ability to delay cataract formation. Following induction of diabetes in young rats by tail vein injection of streptozotocin (STZ), all rats with blood glucose levels >300 mg/dL were divided into four equal groups of eight rats. Group 1 received normal chow (untreated diabetic control), group 2 received chow containing 0.0125% of the aldose reductase inhibitor (ARI) AL1576, group 3 received chow containing 0.05% of compound **4,** and group 4 received chow containing 0.05% of compound **8**. A fifth group was composed of non-diabetic, age-matched control rats. Feeding records indicated that the diabetic rats ingested an average dosage ± S.D. of 48.7±3.5 mg/kg/day of compound **4**, 48.9±2.6 mg/kg/day of compound **8**, and 22.8±2.1 mg/kg/day of AL1576. As summarized in Table 1, hyperglycemia among the diabetic groups did not significantly differ as measured by both blood sugar and glycosylated hemoglobin levels indicating that neither the multi-functional antioxidants nor ARI affected hyperglycemia. Furthermore, treatment with antioxidants did not significantly affect the body weights of the diabetic animals (Figure 7); however, the rats treated with compound **4** appeared on average to eat slightly more during the course of the study and therefore weighed slightly more.

**Table 1:** Level of hyperglycemia in rats.

	Blood Glucose *		HbA1c#
	Day 0	Day 49	Day 49
Non-diabetic Control	197.8±18.8	223.9±15.0	4.8±0.08
Untreated Diabetic	455.4±23.2	600.0±0.0	12.9±0.07
Diabetic + AL1576	508.3±30.2	591.2±8.2	12.8±0.18
Diabetic + **4**	497.3±31.2	600.0±0.0	12.8±0.11
Diabetic + **8**	518.0±27.8	600.0±0.0	12.8±0.14

*Blood sugar levels were determined at the initial start of the study (Day 0) and the end of the study (Day 49). Values read as HIGH (above 600 mg/dL) on the glucometer were averaged as 600. Mean ± SEM; n = 8.

# Glycosylated hemoglobin (HbA1c) levels were also determined at the end (day 49) of the study. Levels >13% were averaged in as 13%. While “stress” can increase blood sugar levels, the HbA1c levels indicate that all diabetic rats were equally hyperglycemic over the study period. Mean ± SEM; n = 8.

**Figure 8:**
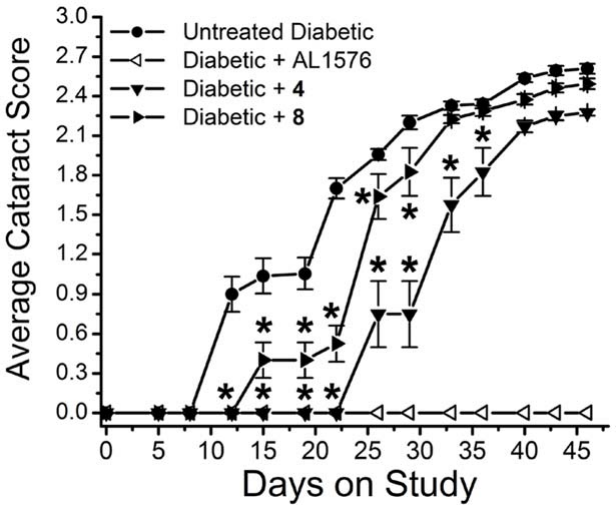
Progression of sugar cataract formation in streptozotocin-induced diabetic rats treated with/without the ARI, AL1576, and multi-functional antioxidants 4 and 8. Values represent mean ± SEM; n = 8. An asterisk (*) denotes a significant difference (p<0.05) when compared to untreated, diabetic rats.

Lens opacities, as monitored by portable slit lamp at 3–4 day intervals, rapidly developed in the untreated, diabetic rats, with vacuole formation detected by 12 days. Treatment with compound **4** and **8** delayed the initial appearance of vacuoles by 26 and 3 days, respectively (Figure 8). Cortical opacities formed by 29 days in all untreated diabetic rats and these were delayed in all **4**-treated and **8**-treated, diabetic rats. At the end of the 7 week study all untreated diabetic rats had developed dense cortical cataracts while those treated with either antioxidant displayed cortical opacities where the posterior capsule was still visible.

**Figure 9:**
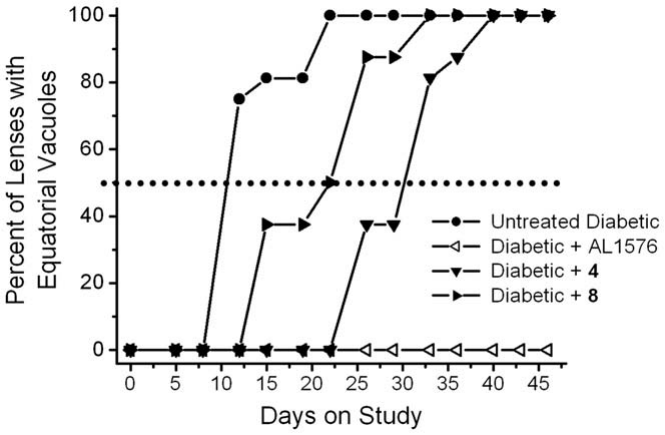
Percentage of animals progressing to equatorial vacuoles (cataract score 1) in diabetic rats treated with/without the ARI, AL1576, or multi-functional antioxidants 4 and 8. Values represent percentage of the 8 animals in each group. Delay was calculated based on the number of days required for 50% of the population to achieve this level of lens opacity (dotted line).

**Figure 10:**
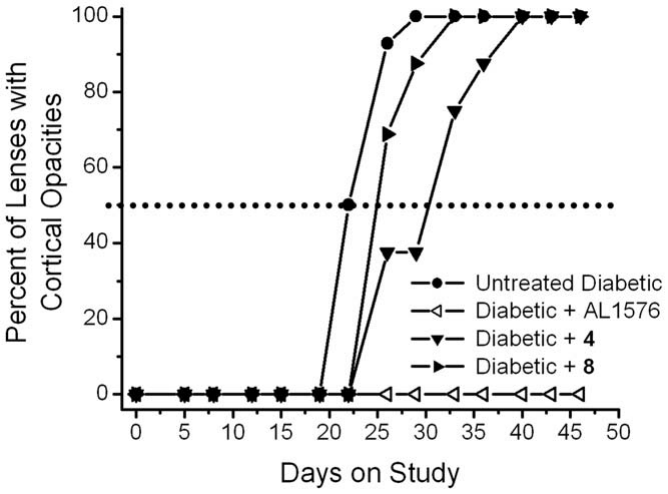
Percentage of animals progressing to cortical opacities (cataract score 2) in diabetic rats treated with/without the ARI, AL1576, or multi-functional antioxidants 4 and 8. Values represent percentage of the 8 animals in each group. Delay was calculated based on the number of days required for 50% of the population to achieve this level of lens opacity (dotted line).

Cataract progression, assessed as the time required for 50% of diabetic rats in each group to develop equatorial vacuoles or cortical opacities, are summarized in Figure 9 and 10. Vacuole formation was delayed by 20 days in **4**-treated rats and 12 days in **8**-treated rats and (Figure 9). A similar trend was observed for cortical cataract formation where treatment with compounds **4** and **8** delayed cortical cataracts by 8 and 3 days, respectively (Figure 10). No lens changes were observed in either the non-diabetic control rats or the diabetic rats treated with the aldose reductase inhibitor AL1576 (Figures 9, 10).

**Figure 11:**
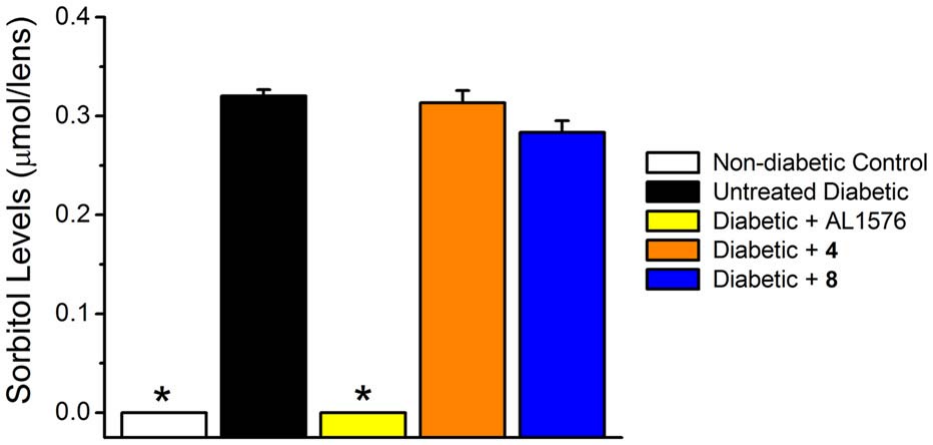
Lens sorbitol accumulation in streptozotocin diabetic rats treated with/without the ARI, AL1576, or multi-functional antioxidants 4 and 8. Values represent mean ± SEM; n = 8. An asterisk (*) denotes a significant difference (p<0.05) when compared to untreated, diabetic rats.

At the end of the study, lenses were removed and analyzed for sorbitol levels according as previously described [34]. Analyses showed that sorbitol accumulation was similar in the untreated diabetic and both multi-functional treated diabetic rats (Figure 11). Sorbitol was absent in the lenses from non-diabetic control rats and diabetic rats treated with the ARI, AL1576. Therefore, the delay in cataract formation by compounds **4** and **8** was not due to AR inhibition. Lens levels of compounds **4** and **8** measured by HPLC-MS were 412±33 ng/mg protein and 206±17 ng/mg protein, respectively (Figure 6B).

### Effect of Compounds 4 and 8 on Endoplasmic Reticulum Stress *In Vitro*

Sorbitol-linked osmotic stress in lens epithelium cells has recently been reported to induce ER stress that leads to the generation of ROS. 

**Figure 12:**
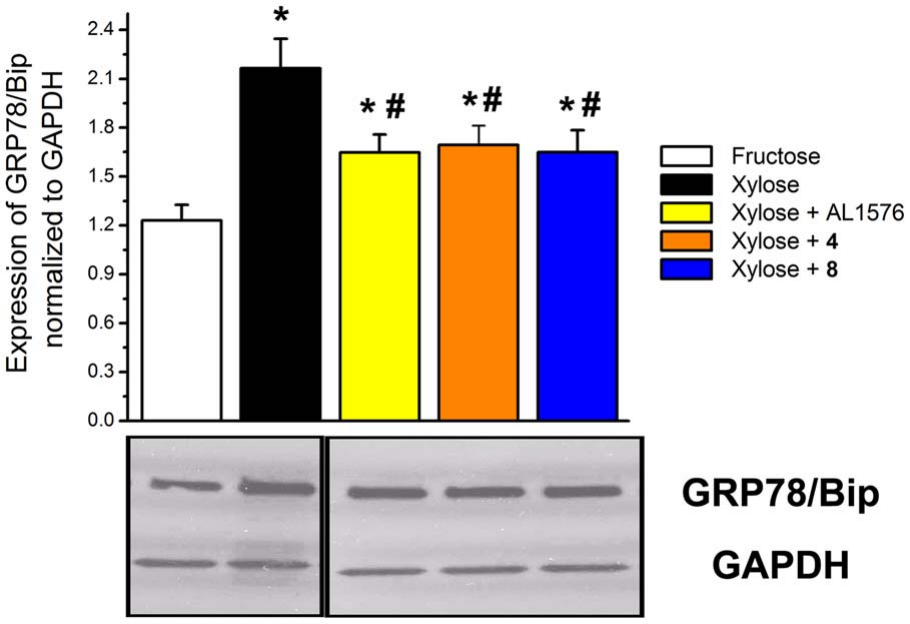
Expression of GRP78/Bip in cultured lenses. Top: Comparison of expression of GRP78/Bip normalized to GAPDH levels in rats lenses cultured in TC-199 bicarbonate media with/without compounds **4,** **8** or AL1576. Lenticular protein homogenate was subjected to Western blotting with anti-GRP78/Bip and anti-GAPDH antibodies. Bottom: Lane 1, media containing 30 mM fructose (control); Lane 2, media containing 30 mM xylose; Lane 3, media containing 30 mM xylose +0.1 mM AL1576; Lane 4, media containing 30 mM xylose media +0.1 mM compound **4**, Lane **5**, media containing 30 mM xylose +0.1 mM compound **8**. Samples were loaded onto the gel in different order than the graph. Therefore, fructose and xylose lanes had to be cut and flipped horizontally using Adobe Photoshop. Pixel intensity was analyzed by ImageJ software. Mean ± S.D.; n = 6. An asterisk (*) denotes a significant difference (p<0.05) when compared to lenses cultured in fructose control media. A pound sign (#) denotes a significant difference (p<0.05) when compared to untreated lenses cultured in 30 mM xylose media.

To determine whether the observed delay of the multi-functional antioxidants in diabetic cataracts is linked to ER stress, the levels of glucose-regulated protein 78/binding immunoglobulin protein (GRP78/Bip), a marker for ER stress, were evaluated in rat lenses cultured *in vitro*. Since opacities do not rapidly form *in vitro* when rat lenses are with glucose, xylose was substituted for glucose because xylose is more rapidly reduced to xylitiol by AR. After 24 hr of culture lens cortical opacities formed in all lenses cultured in 30 mM xylose with/with compounds **4** or **8**; however, opacities were absent in lenses cultured in xylose media containing AL1576. Similarly, control lenses cultured for 24 hr in TC-199 fructose media remained clear. Compared to the control cultured lenses, expression of GRP78/Bip in xylose cultured lenses was significantly induced (Figure 12). This expression was reduced in lenses treated with either compounds **4**, **8**, or AL1576. Since compounds **4** and **8** do not reduce sorbitol by inhibiting AR, their delay of cataract formation must be downstream of the osmotic effect that induces GRP78/Bip and generates ROS.

## Discussion

Age-related cataracts are considered to be multi-factorial in origin, with ROS mediated oxidative damage considered to be a common factor [7], [35], [36]. Iron concentrations also increase in the ageing lens and a major route to damaging ROS formation is through the Fenton reaction, which produces hydroxyl radicals using iron as the catalyst [37]. In the present study two multi-functional antioxidants **4** and **8**, which independently scavenge free radicals and chelate iron [25], [38], [39], [40], [41], [42] have been evaluated for their ability to delay cataract formation in two animal models where oxidative stress has been reported [10], [11], [12], [13], [19], [20].

Rat exposed to at least 2 Gy of whole head gamma radiation form cataracts after a dose-dependent latency period [11], [12], [13]. The deleterious effect of gamma rays on the lens is mediated by the radiolysis of water to hydrogen peroxide (H_2_O_2_), hydroxyl radicals (OH^•^), and superoxide (O_2_^−•^) [14]. Of these reactive oxygen species, the hydroxyl radicals are considered to be the primary initiators of oxidative damage, while superoxide and hydrogen peroxide typically lack the sufficient reactivity to oxidize critical biological macromolecules [15]. Gamma radiation-generated superoxide has also been shown to cause release of iron from ferritin, which can enhance production of hydroxyl radicals through the Fenton and Haber-Weiss reactions leading to DNA, protein, and lipid damage [14].

As expected, 15 Gy of whole head irradiation resulted in damage to the salivary glands. It was noted that rats pre-treated with either compound **4** or **8** lost 4% of their body weights compared to a 10% loss in untreated rats, suggesting that these compounds were radioprotective. Since pantethine has been used as a radioprotectant, these compounds could potentially be co-administered with pantethine to synergistically protect against radiation. However, further studies are required to confirm this observation. Radiation damage to the lens is typically first observed as PSC opacities [43], [44]. In pigmented animals, PSC opacities were observed after a latency period of eight weeks, compared to the 5 weeks typically seen in albino animals [27]. This extended latency period may be due in part to the pigment of the iris, but may also reflect strain differences in the lens, as suggested by observations that lenses from pigmented animals are more tolerant to UV radiation *in vitro* [45]. Initial lens changes appeared as a diffuse PSC clouding (haze) which progressed to dense PSC opacification and punctate opacities that blocked visualization of the retina. At the end of the study, total lens opacification, but not mature cataracts, were present. A number of studies have reported the use of antioxidants and phase transition inhibitors to delay radiation-induced cataracts in albino rats [10], [26], [27], [46], [47], [48], [49], [50]. In the present studies using pigmented rats, treatment with pantethine and multi-functional antioxidants **4** and **8** delayed the appearance of lens opacities. However, the delay by pantethine was minimal, unlike the marked delay observed in irradiated albino animals [26]. The observed delay by the multi-functional antioxidants correlated with the lenticular presence of multi-functional **4** and **8** in the lens.

Another common model for cataract formation is the diabetic rat. Chronic hyperglycemia results in severe metabolic imbalances and non-physiological changes in many tissues, including the formation of a diabetic ‘sugar’ cataract [51], [52]. This process is initiated when glucose is reduced to its sugar alcohol, sorbitol, by the enzyme AR [18]. Hyperosmotic effects result from the accumulation of sorbitol in the lens epithelium and initiate ER stress [19], [20]. As sorbitol accumulates primarily in the epithelium and superficial lens fibers of the diabetic lens, free radical production is increased and natural antioxidant defenses are compromised. This results in increased oxidative stress [21], [22], [23] and apoptotic signaling. Inhibiting sorbitol induced osmotic stress in the rat lens by the administration of ARIs prevents the biochemical changes associated with diabetic cataract formation [18]. Reducing oxidative stress can only delay cataract formation as demonstrated by the antioxidants butylated hydroxytoluene (BHT) [53], [54], Trolox (6-hydroxy-2,5,7,8-tetramethyl-chroman-2-carboxylic acid) [55], and vitamin E [56].

Antioxidants cannot prevent sorbitol accumulation; however, the overall shift of the treated graphs to the right in Figure 8 indicates a delay in cataract progression presumably by reducing the effects of generated ROS. The absence of cataract formation in AL1576-treated animals is consistent with previously published reports on the effects of ARI treatment on ocular pathology in diabetic animals [57]. Compounds **4** and **8** were able to delay cataract formation without affecting blood sugars (Table 1) or lenticular sorbitol levels (Figure 11). The presence of compounds **4** and **8** in the lens strongly suggests that the delays in cataract formation observed in both animal models are linked to their lenticular levels.

These proof of concept studies represent the first step in the potential development of these compounds as therapeutics agents. Clearly, the development of these compounds requires important pharmacokinetic and pharmacodynamic (PK/PD) studies to determine important parameters such as the minimal therapeutic dose (MTD), 50% of the maximal effective dose (ED_50_), the maximum tolerated dose, the lethal dose, and the half life of the drug (t_1/2_). Efficacy studies where the time to catraract formation (pharmacological endpoint) of these compounds at their ED_50_ is determined, requires repeating the present study at a minimum of three additional doses so that a dose-response curve can be constructed for estimating the ED_50_. These dose-response curves would also allow us to estimate the minimal therapeutic dose (MTD). However, such efficacy studies are also directly linked to the turnover of drug in the lens. In the present experiments, similar lens levels of compounds were achieved (Figure 6) despite different doses being administered, This suggests that the lenticular half-life of compounds **4** and **8** is a key factor in determining the MTD.

In the current studies, the approximate 80 mg/kg/day dose used was based on experience where such a dose by a majority of ARIs either prevented or significantly delayed diabetic cataract formation as illustrated by the laboratories of Kador [28], [29], [58] and Kinoshita [59], [60], [61]. Since the synthesis of compounds **4** and **8,** as described by Jin et al. [25], is straight-forward, we believe that the synthetic scheme is amenable to scaled-up GMP production. In the present studies, the study durations versus the amount of synthesized drug on hand limited the doses utilized for each study. Therefore, chow containing 0.05% of each compound was administered to the young rats in the 7 week diabetes study (*ca.* 80 mg/kg/day dose from feeding studies) while chow containing 0.025% were administered to the much larger rats in the 26 week irradiation study (*ca.* 20 g/kg/day dose from feeding studies). Since the pharmacokinetics of the compounds have not been determined, rats orally received compounds mixed in chow rather than receiving a fixed dose by gavage daily to reduce the potential effects of systemic half-life differences. Rats typically eat when active (awake). Therefore, the effects of a potential short half life on biological activity would be more evident in rats receiving a single bolus of 80 mg/kg/day drug than those receiving low doses of drug adding up to 80 mg/kg/day. While clearly additional studies are required to define the pharmacokinetics and specific efficacy of these compounds, the present study presents a proof of concept that multi-functional antioxidants show promise in significantly delaying ROS generated components of cataract development.
